# Hybrid Neural Network Architecture for Automated Liver and Tumor Segmentation Using Ensemble Learning on CT Images

**DOI:** 10.3390/biomimetics11060366

**Published:** 2026-05-25

**Authors:** Maryam Khoshkhabar, Saeed Meshgini, Reza Afrouzian

**Affiliations:** Department of Biomedical Engineering, University of Tabriz, Tabriz 5166616471, Iran

**Keywords:** liver tumor detection, hybrid neural network, ensemble architecture, biological systems inspiration, biomimetic systems

## Abstract

Accurate and automatic segmentation of the liver and liver tumors from computed tomography (CT) images is essential for computer-assisted diagnosis, treatment planning, and clinical decision-making. Although deep learning-based segmentation models, particularly U-Net and its variants, have achieved promising results in medical image analysis, many existing approaches mainly focus on local pixel-level feature extraction and may have limited ability to explicitly model long-range spatial relationships among anatomically meaningful regions. In addition, liver tumor segmentation remains challenging due to low contrast, irregular tumor boundaries, heterogeneous tumor appearances, and noise or artifacts in CT images. To address these limitations, this study proposes a hybrid ensemble neural network architecture that integrates an improved U-Net and a Graph U-Net for automatic liver and liver tumor segmentation. The improved U-Net is designed to capture fine-grained local features and preserve detailed spatial information through an encoder–decoder structure with skip connections, while the Graph U-Net uses Simple Linear Iterative Clustering (SLIC)-based superpixels to construct a graph representation of CT images and model spatial dependencies between adjacent image regions. By combining these complementary representations through an ensemble learning strategy, the proposed framework enhances both pixel-level segmentation accuracy and robustness against noisy imaging conditions. The proposed method was evaluated on the LiTS17 dataset, where CT images were preprocessed using intensity filtering, resizing, data augmentation, and normalization. Experimental results demonstrate that the proposed ensemble architecture achieves 99.2% accuracy for liver segmentation and 98.1% accuracy for liver tumor segmentation, outperforming representative segmentation models such as MultiresUnet and R2U-Net. Furthermore, robustness experiments under different signal-to-noise ratio conditions show that the proposed model maintains stable performance in noisy CT images, achieving 85% accuracy even under severe noise at −4 dB SNR. This result highlights the advantage of integrating convolutional feature learning with graph-based spatial relationship modeling for improving segmentation stability when image quality is degraded by noise or artifacts. These findings indicate that the integration of improved U-Net, SLIC-based graph construction, and Graph U-Net provides an effective and noise-robust solution for liver and liver tumor segmentation, with potential applicability as a computer-assisted tool in clinical image analysis after further validation on larger and external datasets.

## 1. Introduction

The liver, a vital organ located beneath the right ribs and adjacent to the lower lungs, plays a crucial role in various physiological processes, including digestion, nutrient storage, and blood cell filtration [1]. The liver is divided into two main lobes—right and left—with two additional lobes: the caudate and quadrate lobes [2]. Hepatocellular carcinoma, one of the most common forms of liver cancer, arises from the rapid and uncontrolled growth of liver cells. This type of cancer occurs when liver cells begin behaving abnormally, leading to a breakdown in regulation and spreading to other parts of the body [3]. Liver cancer, particularly among men, has become a global health concern, with infection rates approximately double that of women worldwide [4]. According to the World Health Organization (WHO), cancer caused 8.8 million deaths in 2015, with liver cancer contributing significantly to this number, causing 788,000 deaths [5]. The American Cancer Society (ACS) reports that approximately 40,710 new cases of liver cancer and bile duct cancer are diagnosed annually in the United States, with a substantial number of deaths expected from this condition [6]. Liver cancer is especially prevalent in regions such as Sub-Saharan Africa and East Asia, where over 600,000 deaths were attributed to liver cancer in 2017 [7]. Liver cancer not only poses a substantial public health threat but also ranks as one of the leading causes of death in both men and women globally [8]. Various imaging techniques, such as sonography, computed tomography (CT), magnetic resonance imaging (MRI), and positron emission tomography (PET), are employed to detect and classify liver abnormalities and tumors [9]. However, each of these methods has its own strengths and limitations, particularly in terms of speed and accuracy [10]. Among these imaging techniques, CT scanning is widely regarded as one of the most effective methods due to its ability to provide detailed three-dimensional images of the liver’s anatomy [11]. However, despite its advantages, CT imaging presents significant challenges in accurately identifying liver tumors. The variability in liver shape and texture across CT images, coupled with the minimal contrast between the liver and adjacent tissues, complicates the process of tumor detection [12]. Additionally, the presence of artifacts in CT images can obscure liver boundaries, leading to potential misdiagnosis [13]. Given these challenges, the development of automated systems for the identification and segmentation of liver tumors has become essential [14]. Recent advances in machine learning and image processing have led to significant improvements in liver lesion detection from CT images [15]. However, while these methods show promise in controlled environments, their application in real-world clinical settings remains a challenge [16].

Given the importance of automated detection of liver tumors, extensive studies have recently been conducted in this regard. For example, the study by Zheng and colleagues, in terms of using 4D information and combining it with LSTM and CNN models, shows that this approach can be highly effective for detecting and segmenting liver tumors. Although this approach may require more resources and processing time due to computational complexities, it offers higher accuracy in delivering results compared to simpler models [17]. Hänsch et al.’s research shows that while the use of the 3D U-Net architecture has significant advantages, such as the ability to process 3D data and capture more details in the image, there are challenges in practice, such as low classification accuracy and slow segmentation processes, which could limit its applicability in clinical environments [18]. In Ahmad et al.’s research, the fast-processing speed and use of a simple and efficient model make this model suitable for situations where processing time is less critical. However, since the model has lower accuracy than other, more complex models, it may not be suitable for applications that require higher precision [19]. Rahman et al.’s study shows that the combination of ResNet and U-Net networks effectively improves model accuracy and performs very well in tumor segmentation. However, in cases where small or complex tumors are present, this model may need further improvements [20]. Manjunath et al.’s research utilized modified U-Net networks to achieve highly accurate results in liver and liver tumor segmentation. However, this model may perform less well on certain datasets, especially for segmenting more complex tumors [21]. Manghi and colleagues evaluated advanced segmentation models on various liver tumor datasets (LiTS, HCC-TACE-Seg, WAW-TACE). Their work focused on improving automated tumor segmentation for diverse liver tumor shapes and sizes. The study emphasized the effectiveness of deep learning models in tumor localization and diagnosis but highlighted challenges in generalizing across different datasets and tumor types [22]. Rahman and team proposed a hybrid deep learning model combining ResUNet and Inception v4 for liver tumor segmentation in CT images. The model achieved high accuracy (99.27%) and Dice score (98.86%) on the 3D IRCADB01 dataset. However, they noted difficulties in detecting smaller and more complex tumors [23]. Balaguer-Montero introduced SALSA, a fully automated tool for liver tumor detection and segmentation. Evaluated on 1598 CT scans, SALSA achieved 99.65% patient-wise detection precision and a Dice coefficient of 0.760. It outperformed traditional methods and showed potential for clinical use in diagnosis and treatment [24]. Goceri and colleagues proposed a U-Net-based hybrid model integrating residual connections and transformer-based attention for liver and tumor segmentation. Their method improved segmentation performance, especially in complex conditions, by capturing both global context and fine details [25]. Ekşi and team compared U-Net, DeepLabV3+, and SegFormer models for liver tumor segmentation using the LiTS dataset. They found that while U-Net was effective with limited data, transformer-based models like DeepLabV3+ and SegFormer showed superior performance with larger datasets, making them suitable for future clinical applications [26]. Chen et al. proposed SBM–Attention U-Net, a hybrid transformer-based network for liver and liver tumor segmentation in medical images. Their architecture integrates BiFormer into the lower encoder layers of Attention U-Net to strengthen global semantic context modeling and long-range pixel-wise dependencies. In addition, a spatial-channel dual attention mechanism was incorporated into the shallow encoder layers to improve fine-grained boundary representation, while a Mix Structure Block was used in the decoder to enhance the fusion of deep semantic and shallow spatial features. The method was evaluated on multiple public datasets, including 3Dircadb, LiTS, and CHAOS, achieving mean Dice scores of 0.9377, 0.9257, and 0.9611, respectively. Although the model demonstrated strong segmentation performance and effective attention-based feature refinement, its hybrid transformer-attention structure may increase computational complexity and may require further validation in real-time clinical environments [27]. Li et al. introduced MAFA-TransUNet, a multi-scale attention and feature aggregation framework based on Transformer U-Net for liver tumor segmentation. This model was designed to address the limitations of conventional U-Net models in capturing global context and multi-scale information, as well as the high computational burden of pure transformer-based methods. The proposed architecture includes a Multi-scale Fusion and Attention Enhancement module to integrate multi-scale features with spatial, channel, and pixel-level attention, and a Dual-Phase Feature Aggregation module to improve feature integration across different modalities or imaging phases. Experimental evaluations on CT and MRI datasets showed that MAFA-TransUNet achieved superior performance in Dice coefficient, mean Intersection over Union, and mean Hausdorff distance compared with existing methods. However, the model’s dependence on multi-scale and multi-phase feature aggregation may increase architectural complexity and requires further investigation for efficient clinical deployment [28]. Sun et al. proposed LCMambaNet, a clinically oriented 2D liver tumor segmentation framework based on selective state-space models and liver cancer-specific attention. Unlike heavy 3D segmentation architectures, LCMambaNet was designed to balance segmentation accuracy and computational efficiency. The model uses a tailored scan-patch mechanism to extract texture- and density-related features from liver cancer regions, while the Liver Cancer Attention Module is used to reduce the confounding effect between normal liver parenchyma and tumor characteristics. The model was evaluated on the LiTS dataset and the CirrMRI600+ dataset. On LiTS, LCMamba-T achieved a Dice coefficient of 92.94 ± 3.12%, while LCMamba-S showed strong performance for small lesions, indicating its potential for early diagnosis and precise treatment planning. Despite these advantages, the method is mainly based on 2D slice-wise segmentation and may still have limitations in fully exploiting volumetric 3D contextual information [29]. Zhu et al. developed HMC-Transducer, a hierarchical Mamba-CNN transducer for robust 3D liver tumor segmentation. The model was proposed to overcome the trade-off between CNN-based local feature extraction and transformer-based global context modeling, particularly in high-resolution 3D CT volumes where transformer complexity becomes computationally expensive. HMC-Transducer integrates CNN modules with linear-complexity Mamba-based long-range modeling through a Direction-Aware 3D Mamba block and a gated Mamba-CNN transducer module. The model was evaluated on multiple public benchmarks, including LiTS17, MSD-Liver, and KiTS21, and demonstrated strong segmentation accuracy, computational efficiency, and generalization ability. On the combined LiTS17 and MSD-Liver evaluation, the method achieved a Dice similarity coefficient of 89.67 ± 1.25%, HD95 of 12.11 ± 2.05 mm, and ASSD of 0.98 ± 0.10 mm. Although the method showed strong robustness and generalization, its 3D architecture may still require relatively high memory resources compared with lightweight 2D approaches [30].

Despite the recent progress in liver and liver tumor segmentation using deep learning-based methods, several research gaps still remain. First, most CNN-based and U-Net-based architectures mainly focus on local pixel-level feature extraction and may not sufficiently capture long-range spatial dependencies among anatomically meaningful regions in CT images. Second, although transformer-based and attention-based methods have improved global context modeling, they usually require large-scale training data and high computational resources, which may limit their applicability in real-time or resource-constrained clinical environments. Third, graph-based representations have shown potential for modeling irregular anatomical structures; however, their integration with conventional convolutional segmentation networks for liver tumor segmentation remains relatively underexplored. Fourth, many existing studies mainly report segmentation accuracy under normal imaging conditions, while robustness against noisy CT images and imaging artifacts is not sufficiently investigated. Finally, comparative evaluations in several previous works are often limited to a small number of baseline models, making it difficult to clearly assess the progressive improvement of newly proposed methods over recent state-of-the-art approaches. Therefore, there is still a need for a segmentation framework that can jointly capture local pixel-level details, model spatial relationships between image regions, maintain robustness under noisy conditions, and provide competitive performance compared with recent high-performing segmentation models.

The motivation for combining an improved U-Net with a Graph U-Net is directly related to the limitations observed in previous segmentation models. U-Net-based architectures are highly effective in preserving local spatial information and extracting pixel-level boundary features through encoder–decoder structures and skip connections. However, because they mainly operate on regular pixel grids, they may be sensitive to local intensity variations, noise, and weak tumor boundaries, which can reduce DSC values, particularly in small or irregular tumor regions. In contrast, Graph U-Net represents the image at a region level by using SLIC-derived superpixels and graph connections, allowing the model to capture spatial dependencies between anatomically meaningful regions rather than relying only on local pixel neighborhoods. This region-level graph representation can reduce the influence of local noise and improve the consistency of segmentation in complex or low-contrast areas. Therefore, the integration of improved U-Net and Graph U-Net is expected to jointly improve boundary precision, spatial consistency, DSC performance, and robustness under noisy CT imaging conditions.

To address the identified research gaps, this study proposes a hybrid ensemble architecture that integrates an improved U-Net and a Graph U-Net for automatic liver and liver tumor segmentation in CT images. The improved U-Net is designed to capture fine-grained local, boundary-related, and pixel-level features, whereas the Graph U-Net employs SLIC-based superpixel graphs to model spatial relationships between anatomically meaningful image regions. By integrating these complementary representations through an ensemble learning strategy, the proposed framework aims to improve segmentation accuracy, robustness, and generalization, particularly under noisy CT imaging conditions.

The main novelty of this study lies in the joint exploitation of convolutional feature learning and graph-based spatial relationship modeling within a unified ensemble segmentation framework. A key architectural innovation of the proposed method is the transformation of CT images into SLIC-based superpixel graphs and their subsequent processing by a Graph U-Net. Unlike conventional U-Net-based or convolutional models that mainly operate on regular pixel grids and local receptive fields, the proposed framework groups neighboring pixels with similar intensity and spatial characteristics into anatomically meaningful superpixels. In this representation, each superpixel is treated as a graph node, while adjacency relationships between neighboring superpixels are modeled as graph edges. This SLIC-to-Graph U-Net design enables the network to capture intricate and irregular spatial boundaries, as well as region-level structural dependencies, that may be missed by traditional convolutional networks. As a result, the proposed framework is better suited for accurate segmentation of liver tumors with weak contrast, heterogeneous appearance, and complex margins.

The main contributions of this study are summarized as follows:

1. A hybrid ensemble neural network architecture is developed for automatic liver and liver tumor segmentation in CT images by integrating an improved U-Net and a Graph U-Net within a unified framework.
2. A SLIC-based graph construction strategy is introduced to convert CT images into superpixel-level graph representations, where each superpixel is treated as a graph node and adjacency relationships are modeled as graph edges. This enables the Graph U-Net to capture irregular spatial boundaries and region-level anatomical dependencies that may be missed by conventional convolutional networks.
3. The improved U-Net component extracts fine-grained local, boundary-related, and pixel-level features, while the Graph U-Net component models spatial dependencies among irregular image regions; their ensemble combination provides complementary representations for more accurate segmentation.
4. The proposed framework is evaluated under both normal and noisy CT imaging conditions with different SNR levels, demonstrating improved robustness compared with the individual network components.
5. A progressive comparative evaluation is conducted against representative and recent segmentation models, including U-Net-based, residual, multi-resolution, graph-based, and attention-related approaches, to demonstrate the effectiveness and competitiveness of the proposed architecture.

The structure of the rest of the paper is outlined as follows: Section 2 reviews the dataset and the mathematical theory behind graph convolutional and U-Net networks. Section 3 introduces the proposed methodology. Section 4 presents an in-depth analysis of the simulation results obtained using the proposed approach, while Section 5 summarizes and concludes the study.

## 2. Materials and Methods

This section begins with an introduction to the dataset used for liver and liver tumor segmentation. It then explains the mathematical principles of Graph Convolutional Networks (GCNs), which are employed to model spatial relationships between different regions in liver and tumor images. Next, the Simple Linear Iterative Clustering (SLIC) algorithm for generating superpixels and constructing the graph structure is introduced. Finally, the U-Net architecture and the improvements made to it for this task are discussed.

### 2.1. LiTS17 Database

**Figure 1:**
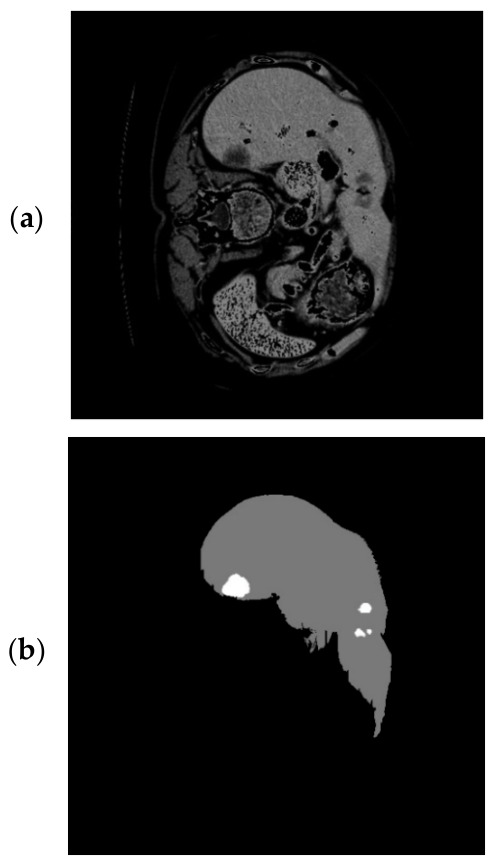
An illustration from the LiTS17 database depicting a CT image, where panel (**a**) presents the original CT scan, and panel (**b**) showcases the associated mask indicating the liver and tumor regions [31].

The dataset used in this study is the LiTS17 (Liver Tumor Segmentation 2017) database [31], which includes data from 130 patients. Each patient has a maximum of 623 CT slices. For the purpose of this research, CT volumes from 10 patients were selected. These volumes contain varying numbers of slices, and only those slices that contain liver tissue were used for liver segmentation. In total, nine volumes from the LiTS17 database were employed in this study. After processing the .nii files, a total of 4158 images, along with their corresponding masks, were available. The liver masks were extracted from these images, and ultimately, 987 images were chosen for training the network. Each image has dimensions of 512 × 512. An example of a CT image with its corresponding tumor and liver mask is shown in Figure 1. It should be noted that this study uses a selected subset of the LiTS17 dataset rather than the full LiTS17 cohort. The subset was selected to conduct an initial evaluation of the proposed hybrid ensemble architecture under controlled experimental conditions. Although the selected images include liver and tumor regions and allow the proposed method to be evaluated for segmentation performance, the limited number of cases may restrict the generalization ability of the model. Therefore, the results should be interpreted as a preliminary evaluation of the proposed framework, and further experiments on the complete LiTS17 dataset and additional external datasets are required.

### 2.2. Overview of the Graph Convolutional Network Model

The foundational concept of GCN was introduced by Michael Deferard and his team in 2016. This work marked the first application of signal processing and graph spectral theory to graphs, which enabled the derivation of convolutional functions and the use of convolutional networks within the framework of graph theory. In graph theory, two key matrices, the adjacency matrix and the degree matrix, play a crucial role. The adjacency matrix defines the connections between vertices in the graph, while the degree matrix can be derived from it. The diagonal elements of the degree matrix represent the sum of the edges connecting to each corresponding vertex. This degree matrix can be expressed as **D**, and the graph matrix as **A**, with the *i*-th diagonal element of the degree matrix defined as follows:

(1) $$D_{ii}=\sum_{j}A_{ij}$$

The Laplacian matrix is defined by the following relationship:

(2) L=D−A

This equation shows that the Laplacian matrix is obtained by subtracting the adjacency matrix from the degree matrix, and it is essential for calculating graph basis functions. These functions can be derived via Singular Value Decomposition (SVD) applied to the Laplacian matrix. Additionally, the Laplacian matrix can be expressed using the eigenvector matrix and singular value matrix, as represented in the following Equation (3):

(3) $$L=U\Lambda U^{T}$$

where **U** denotes the matrix of eigenvectors, and **Λ** is the matrix of eigenvalues.

Furthermore, the Fourier transform of a graph signal can be expressed in terms of the eigenvectors, with Fourier bases defined by the diagonal eigenvalues, **λ**, as shown in the relation below:

(4) $$f(\lambda )=U^{T}f$$

To aid in understanding, the Fourier transform and its inverse for a signal like *f* can be expressed in Equations (5) and (6), respectively:

(5) $$F=U\Lambda U^{T}f$$

(6) $$f=U\Lambda^{-1}U^{T}F$$

Equation (5) represents the Fourier transform of the graph, while Equation (6) illustrates the feature vector of a signal in the context of Fourier bases and the graph’s Fourier transform. The graph convolution operator can also be computed by performing the convolution of two signals within the graph domain using the Fourier transforms of each signal. The convolution of two signals, *z* and *y*, with the operator is defined as follows:

(7) $$(Z*Y)(\lambda )=U^{T}z.U^{T}y$$

In this equation, the filter function is used to define the graph convolution operator when integrated with neural networks. Thus, *z* is the filtered version of the signal *y*:

(8) z=W∗y

Finally, by decomposing the Laplacian matrix into its singular values and eigenvectors, graph convolution is formally defined as [32,33,34]:

(9) $$z=\sum_{i}\Lambda_{i}U_{i}^{T}y$$

### 2.3. General Model of SLIC Algorithm

The SLIC algorithm is employed to divide the input image *I* into several distinct regions, forming an adjacency graph *G*. The number of regions, denoted as *k*, is determined randomly, while the spatial resolution is defined by the product *P* × *Q*, where *P* and *Q* represent the image’s dimensions. The image features are normalized to the range [0, 1] by dividing each channel by the bit depth *B*, which corresponds to the number of bits used for each color channel [35].

In the resulting neighborhood grid, superpixels *R*_*i*_ are represented as vertices *V*_*i*_, each associated with a one-dimensional feature vector *F*_*i*_ that reflects the characteristics of the corresponding region. The average pixel intensity for each superpixel *R*_*i*_ is computed by taking the mean intensity of all pixels within the region. Adjacent superpixels are linked by weighted edges, where the weights are determined using a Gaussian weighting function. The weight *W*_*ij*_ between two adjacent regions is calculated as:

(10) $$W_{ij}=\exp(-\frac{d^{2}}{2\sigma^{2}})$$

where *d* is the spatial distance between regions, and *σ* is a parameter that controls the sensitivity of the weight.

The adjacency matrix *A*, with size *N* × *N*, captures the relationships between regions and is given by:

(11) $$A=[a_{ij}]\in {\mathbb{R}}^{N\times N}$$

Additionally, there is a region feature matrix $X\in {\mathbb{R}}^{N\times 1}$, which holds the features of the *N* vertices in the graph, with each vertex representing the characteristics of the respective region [36].

### 2.4. Overview of the U-Net Networks

U-Net networks are widely used in medical image segmentation, particularly for simulating and extracting precise features in both 2D and 3D medical images. These networks were originally developed by Olaf Ronneberger, Philipp Fischer, and Thomas Brox for segmenting biological cells in microscopy images, but quickly expanded to other medical fields, such as organ and tissue segmentation in CT and MRI scans [26].

The architecture of U-Net consists of two main parts: an encoder and a decoder. The encoder is primarily responsible for extracting features from the input image, while the decoder attempts to reconstruct these features into the final image that includes the segmented regions. One of the key features of this architecture is the use of skip connections, which link the encoder and decoder. These connections transmit finer details from the earlier encoder layers to the later decoder layers, helping to preserve image details and improve segmentation accuracy.

The core concepts that enable the U-Net architecture to perform well in medical image processing include the ability to learn features at different scales, detect and separate various structures in complex images, and maintain precise spatial information through skip connections. These features allow the network to achieve high accuracy in segmentation, even in the presence of noise or low-quality images. In the field of medical image segmentation, U-Net architecture has become a popular choice for deep learning-based models due to its suitability for problems that require maintaining spatial details. These features have made U-Net one of the primary tools used in artificial intelligence systems for medical applications such as tumor detection, tissue simulation, and internal body structure analysis [36].

## 3. The Suggested Model

This section describes the proposed model of this study, which includes data preprocessing, the use of graph architecture, SLIC formation, and the use of UNet for automatic detection of liver tumors.

### 3.1. Pre-Processing Stage

In this section, the preprocessing used in the present study is presented. For this purpose, the pixel intensity values, represented by Hounsfield units, undergo preprocessing using a bandpass filter with a range of [0, 150] to eliminate noise and irrelevant intensity values. After the resizing process during preprocessing, the images are standardized to a dimension of 256 × 256. To further enhance the dataset, image augmentation is applied, including horizontal and vertical flipping as well as rotations within a range of 0 to 30 degrees, thus increasing both the number of images and their corresponding mask images. The final step in the preprocessing pipeline involves normalization, where the intensity values are scaled to a range between 0 and 1.

### 3.2. Graph SLIC Stage

In our proposed method, we use the SLIC (Simple Linear Iterative Clustering) technique to obtain a graph-based representation of the image. This approach divides the image into distinct regions, called superpixels, where each superpixel represents a specific area of the image. These superpixels are formed by clustering pixels that are similar in both color and spatial proximity.

In this process, each superpixel is treated as a node in the graph, and the connections between nodes (edges) are defined based on the proximity and similarity of their respective regions. For each superpixel, we calculate a feature vector that represents the average pixel intensity within that region. This feature vector helps to model and characterize the properties of each node in the graph. Next, we create the edges of the graph by assessing how close the superpixels are to each other. If two superpixels are adjacent, an edge is created between them. Non-adjacent regions are not connected. Finally, an adjacency matrix is generated to represent these connections, which captures the relationships between superpixels and is used for further analysis in our proposed method.

### 3.3. Proposed Deep Ensemble Network

**Figure 2:**
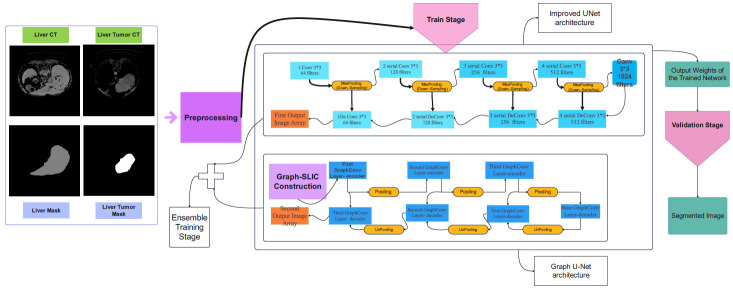
The proposed ensemble architecture.

The schematic overview of the proposed method is illustrated in Figure 2. In this approach, the CT images from the LiTS17 database are utilized as the primary dataset for the automatic detection of both the liver and liver tumor lesions. As shown, the method begins with a preprocessing stage where the raw CT images are processed to enhance their quality and reduce noise. Once preprocessed, the obtained images are passed through the proposed ensemble architecture for further analysis.

During the training phase, the network utilizes both the CT images and their corresponding ground truth masks, which delineate the liver and tumor regions. This enables the network to learn to accurately segment the target structures. The training is performed using an ensemble learning technique, where multiple models are combined to improve performance and robustness. Specifically, the proposed network architecture integrates an improved version of the U-Net model and a Graph U-Net model, creating a hybrid architecture that takes advantage of both frameworks.

In the proposed ensemble architecture, the outputs of the improved U-Net and Graph U-Net are integrated through an output-level weighted fusion strategy rather than a voting-based mechanism. Both subnetworks generate segmentation probability maps with the same spatial resolution as the input image. Let *P_U_* and *P_G_* represent the probability maps predicted by the improved U-Net and Graph U-Net branches, respectively. The final ensemble probability map *P_E_* is computed as:

(12) $$P_{E}=\alpha P_{U}+(1-\alpha )P_{G}$$

where *α* denotes the fusion coefficient for the improved U-Net output and *1 − α* denotes the fusion coefficient for the Graph U-Net output. The value of α is selected based on the validation performance to balance the contribution of local pixel-level features and graph-based spatial features. The final binary segmentation mask is then obtained by applying a threshold to the fused probability map.

To jointly optimize the two branches, a multi-term loss function is used. Each branch is supervised independently, and the fused ensemble output is also optimized against the ground-truth mask. Dice loss is adopted as the primary loss function due to its effectiveness in handling class imbalance in medical image segmentation. The overall objective function is defined as:

(13) $$L_{total}=\lambda_{U}L_{Dice}(P_{U},Y)+\lambda_{G}L_{Dice}(P_{U},Y)+\lambda_{E}L_{Dice}(P_{E},Y)$$

where *Y* is the ground-truth segmentation mask, and *λ_U_*, *λ_G_*, and *λ_E_* are weighting coefficients for the improved U-Net loss, Graph U-Net loss, and ensemble loss, respectively. During training, the total loss is minimized using backpropagation, and the parameters of both subnetworks are updated jointly. This optimization strategy ensures that each branch learns useful segmentation representations while the final ensemble output is directly optimized for accurate liver and tumor segmentation.

The learning of the network’s weights is achieved by minimizing the combined cost functions corresponding to each of the two networks. This dual-cost function approach ensures that both the U-Net and Graph U-Net components are optimized simultaneously, promoting effective learning for liver and tumor segmentation. Additionally, the output generated during the SLIC stage, which involves segmenting and clustering the image into superpixels, is fed into the Graph U-Net component of the architecture. This integration of SLIC-derived superpixels into the Graph U-Net model enhances the network’s ability to capture spatial relationships between adjacent regions, resulting in more accurate segmentation of the liver and its lesions. The final output is a fully trained network capable of accurately extracting and delineating the target organ and its associated tumor regions from CT scans.

#### 3.3.1. Improved U-Net Part of the Proposed Architecture

**Figure 3:**
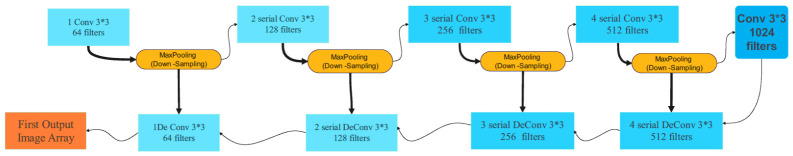
The improved Unet part of the proposed network architecture.

The improved U-Net module, as shown in Figure 3, plays a critical role in the proposed ensemble architecture by extracting local, boundary-related, and pixel-level features from CT images. Similar to the original U-Net, this module follows an encoder–decoder structure with skip connections. However, compared with the original U-Net architecture, the improved U-Net used in this study is modified to enhance hierarchical feature extraction for liver and liver tumor segmentation. The main improvement is the progressive deepening of the encoder path, where the number of consecutive convolutional layers increases across different encoding stages. Specifically, the forward path includes one, two, three, and four serial convolutional layers in successive stages, allowing the network to learn increasingly complex feature representations.

In the shallow encoder layers, the network captures low-level texture, edge, and boundary-related information, which is important for identifying liver contours and tumor margins. In deeper layers, the network extracts more abstract semantic representations that help distinguish liver and tumor regions from surrounding tissues. The number of filters is also increased progressively across the encoder stages, enabling the model to capture richer feature representations at different levels of abstraction. The convolutional design is based on repeated 3 × 3 convolutional kernels. This kernel size provides an effective balance between local feature extraction and computational efficiency. Moreover, stacking multiple 3 × 3 convolutional layers enlarges the effective receptive field while requiring fewer parameters than using larger convolutional kernels. Max-pooling operations are applied in the forward path to gradually reduce the spatial resolution and increase the receptive field, allowing the model to learn higher-level contextual information.

The decoder path uses transposed convolutional layers to progressively recover the spatial resolution of the feature maps and reconstruct the final segmentation mask. Skip connections between the encoder and decoder are preserved and used to concatenate encoder features with the corresponding up-sampled decoder features. This concatenation transfers fine-grained spatial details from the encoder to the decoder, which is essential for accurate delineation of liver boundaries and small or irregular tumor regions. The configuration with four forward-path steps and four max-pooling operations was selected to balance semantic feature abstraction and spatial detail preservation. Since the input CT images are resized to 256 × 256, four downsampling stages reduce the feature map to a sufficiently compact representation while still preserving meaningful spatial information for reconstruction. Using fewer downsampling stages may limit the receptive field and reduce the ability of the model to capture global anatomical context, whereas using more downsampling stages may excessively reduce the feature-map resolution and lead to the loss of small tumor details. Therefore, the four-step configuration provides a reasonable architectural trade-off between contextual representation, boundary preservation, and computational cost. This design choice is further supported by the experimental comparison of different forward-path steps presented in the Results section.

#### 3.3.2. Graph U-Net Part of the Proposed Ensemble Network

**Figure 4:**
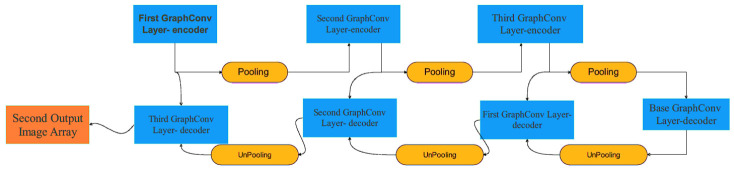
The specifics of the Graph U-Net component within the proposed ensemble network.

The Graph U-Net component of the proposed ensemble network, illustrated in Figure 4, integrates graph-based operations to model spatial relationships between different regions of the image more effectively. This module incorporates graph convolutional layers, which operate on the nodes (representing image regions or superpixels) and the edges (representing the spatial connections between these regions). Through the use of graph convolution, the network effectively captures both local and global dependencies within the image, enhancing its capacity to segment intricate structures, such as the liver and tumors. This spatial modeling is particularly important for handling irregular shapes and complex anatomical features within medical images.

In the forward path of the Graph U-Net, graph pooling layers are employed to down-sample the graph by reducing the number of nodes. This down-sampling process focuses the network on the most important features of the image, enhancing the model’s ability to identify large-scale structures like the liver and tumor. It also helps to reduce the computational load, making the network more efficient. On the other hand, the graph un-pooling layers in the backward path serve to up-sample the feature maps, gradually reconstructing the image to its original size while preserving the spatial relationships learned during the down-sampling process. This step ensures that the output image maintains the correct dimensions for comparison with the target mask image.

Both the forward and backward paths of the Graph U-Net consist of three steps. In each of these steps, the network progressively refines its feature maps. At the end of the forward path, a base graph convolutional layer is applied, followed by graph pooling, which further reduces the spatial dimensions of the image. In the backward path, the un-pooling layers are responsible for restoring the original image dimensions. Additionally, the feature maps from the encoder path (which include the original image features) are concatenated with the up-sampled outputs of the decoder, forming the final segmented image.

Both the improved U-Net and Graph U-Net modules are characterized by a variety of layer types and parameter configurations, which are essential for their performance. Table 1 provides detailed information regarding the Graph U-Net, including the number of layers, weight dimensions, and the operations involved, such as pooling and un-pooling. Graph convolutional layers play a pivotal role in capturing the spatial relationships between regions, while batch normalization is applied to stabilize the training process, ensuring smoother and faster convergence. Table 2 outlines the architecture of the improved U-Net, detailing the convolutional and de-convolutional layers, kernel sizes, number of filters, and activation functions used (such as ReLU). This configuration enables the network to effectively extract low-level features and progressively refine them throughout the layers, ensuring the segmentation process is both precise and efficient.

The integration of the improved U-Net and Graph U-Net in the proposed ensemble architecture allows the network to leverage both traditional convolutional techniques and advanced graph-based methods. The U-Net component excels at extracting local features through its encoder–decoder structure, making it highly effective in identifying key anatomical structures. Meanwhile, the Graph U-Net enhances the model’s ability to capture spatial dependencies across different regions, leading to more accurate segmentation of complex structures such as the liver and tumors. By combining these two powerful architectures, the proposed network provides a robust solution for accurately detecting and delineating liver lesions in CT images, ultimately offering enhanced performance in medical image segmentation tasks.

### 3.4. Training and Evaluation

During the training process, key parameters, including the optimizer, learning rate, number of layers, and loss function, must be carefully selected and fine-tuned. The optimizer governs how the model’s weights are updated, while the learning rate defines the step size for these updates. The number of layers influences the model’s capacity, and the loss function, such as Dice Loss, evaluates the accuracy of the segmentation.

The hyperparameter tuning process was performed using a controlled grid-search strategy combined with validation-based model selection. In this process, several candidate values were evaluated for the main training and architectural parameters, including learning rate, batch size, number of training epochs, optimizer type, number of graph convolutional layers, number of SLIC superpixels, and the number of forward-path steps in the improved U-Net. For each configuration, the model was trained on the training subset and evaluated on the validation subset using Dice coefficient and segmentation accuracy as the main selection criteria. The configuration that achieved the best validation performance while maintaining stable convergence and reasonable computational cost was selected as the final setting.

Specifically, the learning rate was tuned over a range of small values to identify a stable convergence behavior, while the batch size was selected by considering both validation performance and GPU memory limitations. The number of training epochs was determined by monitoring the convergence of Dice loss and validation accuracy. The number of SLIC superpixels and the number of forward-path steps in the improved U-Net were selected based on the comparative experiments reported in the Results section. Similarly, the number of graph convolutional layers was tuned to balance spatial relationship modeling and the risk of over-smoothing in graph representations. The final selected hyperparameters are summarized in Table 3.

Training is conducted using 10-fold cross-validation, where the dataset is divided into 10 subsets, and the model is trained and evaluated on different portions to ensure robustness and assess its generalization capabilities.

## 4. Results

This section presents the simulation results of the proposed Cheb-MA framework, which was implemented on a laptop featuring 16 GB of RAM and a 3.2 GHz Core i7 CPU. The results highlight the effectiveness of the proposed ensemble architecture for liver and liver tumor segmentation, demonstrating its robustness under various conditions, including noisy CT images.

This section is organized into two distinct subsections to separate objective result presentation from interpretive discussion. Section 4.1. Quantitative Segmentation Performance, presents the experimental findings, including the main segmentation results, evaluation metrics, and hyperparameter sensitivity analysis. Section 4.2. Discussion, contextualizes these findings by interpreting the performance of the proposed hybrid ensemble framework, evaluating its noise resilience, comparing the results with existing literature, and discussing clinical relevance, limitations, and future research directions.

### 4.1. Quantitative Segmentation Performance

**Figure 5:**
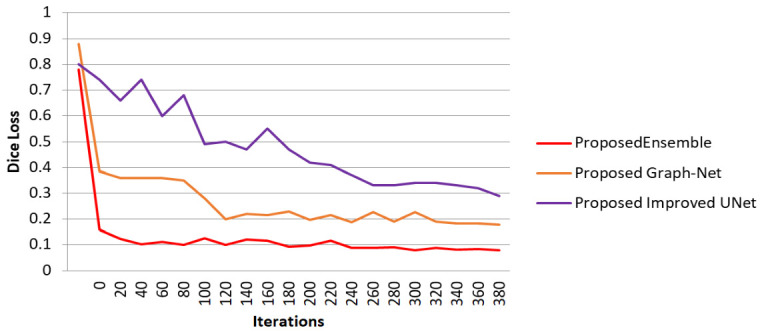
Dice Loss fluctuations for liver segmentation.

The Dice Loss function is a critical metric for evaluating segmentation performance, especially in medical imaging. Figure 5 presents the fluctuation of Dice Loss values across iterations for the liver segmentation task. As observed, the proposed ensemble network requires over 380 iterations to converge, which indicates the complexity and gradual optimization of the model. The plot also compares the performance of three distinct methods: the proposed ensemble, proposed Graph U-Net, and proposed improved network. These results highlight the superiority of the ensemble approach in achieving a more consistent and lower Dice Loss compared to other models.

**Figure 6:**
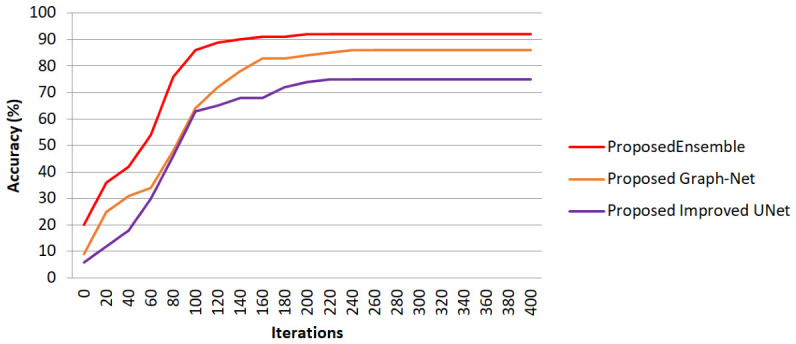
Accuracy fluctuations for liver segmentation.

In Figure 6, we present the accuracy fluctuations over training iterations for the liver segmentation task. This figure, along with the Dice Loss fluctuations, further validates the efficiency of the proposed ensemble network. The proposed ensemble model consistently outperforms the individual models, indicating its superior ability to generalize and segment the liver accurately across diverse test images.

**Figure 7:**
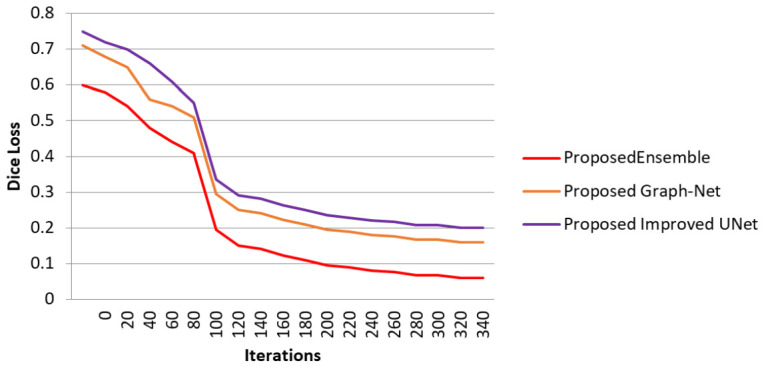
Accuracy fluctuations for liver segmentation.

**Figure 8:**
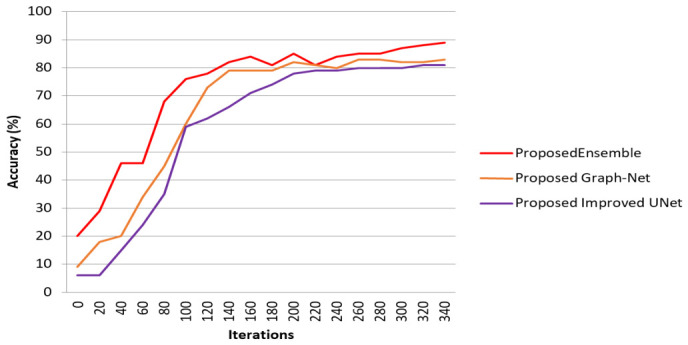
Accuracy fluctuations for liver tumor segmentation.

The performance of the proposed methods for liver tumor segmentation is demonstrated in Figure 7 and Figure 8. The Dice Loss curve for liver tumor segmentation, shown in Figure 7, indicates that the ensemble network converges after approximately 340 iterations, with a significant reduction in loss compared to the other models. Furthermore, the accuracy of the segmentation, illustrated in Figure 8, corroborates the effectiveness of the proposed ensemble method. It demonstrates the ensemble model’s superior ability to detect and segment liver tumors, reflecting its robustness and efficiency in challenging segmentation tasks.

**Figure 9:**
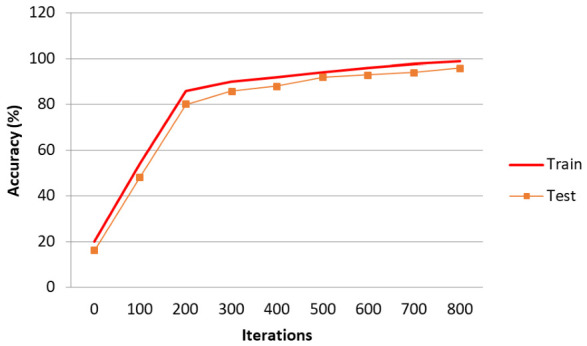
Accuracy fluctuations for liver tumor segmentation.

**Figure 10:**
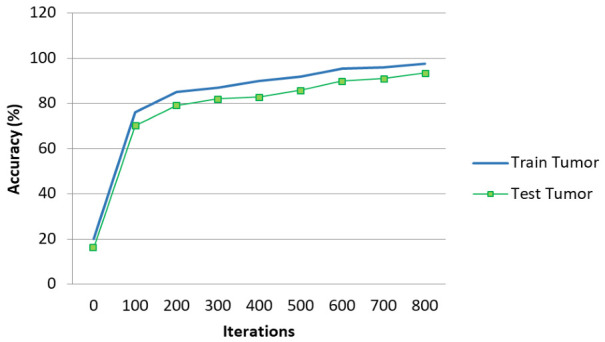
Train and test accuracy of the proposed ensemble for liver tumor segmentation.

Figure 9 and Figure 10 show the train and test accuracy for both liver and liver tumor segmentation. These figures highlight the high performance and consistency of the proposed ensemble network in both training and testing phases. The results confirm that the model performs well not only on training data but also on unseen test data, which is essential for real-world applications where generalization is critical.

In Table 4, we present the segmentation results for liver and liver tumor segmentation. The accuracy, sensitivity, Dice coefficient, and mean-IoU metrics for both liver and liver tumor segmentation are provided. The results demonstrate that the proposed ensemble method achieves outstanding performance, with accuracy values of 99.2% for liver segmentation and 98.1% for liver tumor segmentation, accompanied by high Dice coefficients and mean-IoU scores, highlighting the method’s reliability and precision.

Table 5 provides the performance metrics of the proposed ensemble architecture with varying numbers of SLIC for graph construction. The results demonstrate that the model maintains high performance across different numbers of SLIC values. Notably, increasing the number of SLIC enhances the accuracy and Dice coefficient, with optimal values observed at 20 and 30 SLICs, confirming the effectiveness of the graph-based approach in capturing fine-grained features.

**Figure 11:**
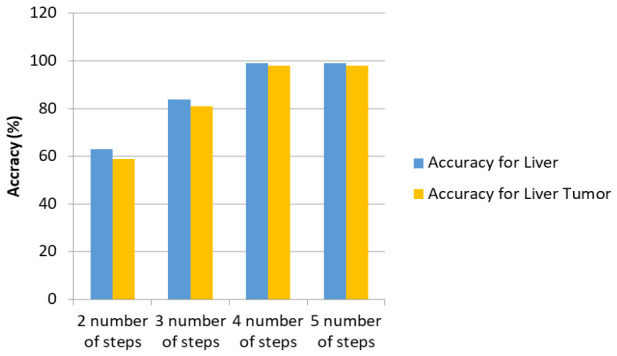
Accuracy comparison for different number of steps in forward path of the improved U-net part of the proposed architecture.

Figure 11 presents a comparison of accuracy for different numbers of steps in the forward path of the U-Net part of the architecture. The optimal configuration is found to be 4 steps, including 4 max-pooling operations for down-sampling, which helps the network focus on the target mask area. This configuration provides the best trade-off between computational efficiency and segmentation accuracy.

To further evaluate the stability of the proposed model, an additional hyperparameter sensitivity analysis was conducted, as shown in Table 6. In addition to analyzing the effects of the number of SLIC superpixels and the number of forward-path steps in the improved U-Net, we investigated the influence of several key training and architectural hyperparameters, including learning rate, batch size, number of training epochs, and number of graph convolutional layers. The purpose of this analysis was to determine whether the proposed ensemble framework maintains stable segmentation performance under different parameter settings. As summarized in Table 6, the proposed model remains relatively stable within a reasonable range of hyperparameter values. The best performance was obtained when the learning rate was set to 1 × 10^−4^, the batch size was set to 8, the number of training epochs was set to 100, and three graph convolutional layers were used in the Graph U-Net component. A smaller learning rate slowed convergence, while a larger learning rate caused less stable optimization. Similarly, a very small batch size increased training fluctuation, whereas a larger batch size slightly reduced the model’s ability to generalize. Increasing the number of graph convolutional layers improved spatial relationship modeling up to three layers; however, further increasing the depth did not lead to additional performance improvement and may introduce over-smoothing in graph representations.

### 4.2. Discussion

**Figure 12:**
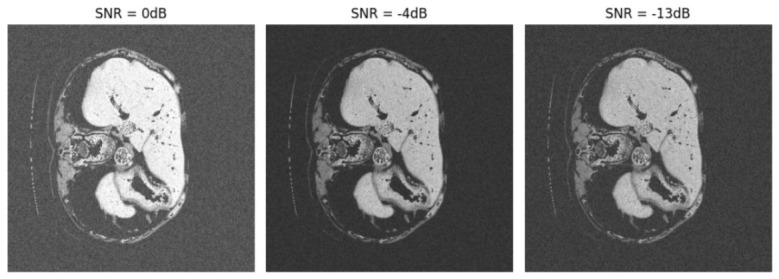
The noisy CT slice with different SNRs.

In Figure 12, the effects of noise on CT images are demonstrated, with the segmentation results provided in Table 7. The table presents segmentation performance across different SNR (Signal-to-Noise Ratio) conditions, from −4 dB to 10 dB. These results highlight the resilience of the ensemble network, as it maintains high segmentation accuracy even under noisy conditions. For example, the proposed ensemble network achieves 85% accuracy at −4 dB SNR and 99.2% in noise-free conditions, illustrating its robustness in handling noisy medical images.

To provide a more comprehensive comparative evaluation, Table 8 was revised and expanded to include both conventional baseline methods and recent representative liver and liver tumor segmentation architectures. In addition to earlier U-Net-based models such as MultiresUnet, SLNet, and R2U-Net, the revised comparison includes more recent methods, including UNet++-based models, attention-based U-Net variants, Swin-UNet-based architectures, residual multi-scale attention U-Net models, and the top-performing algorithms reported in the LiTS benchmark. These methods were selected because they represent major directions in medical image segmentation, including multi-resolution feature extraction, nested skip connections, attention-guided feature refinement, transformer-based global context modeling, residual learning, and multi-scale representation learning. Since the compared studies were conducted using different datasets, preprocessing strategies, train/test splits, and evaluation protocols, the comparison includes the metrics reported in the corresponding original studies, including accuracy, sensitivity, Dice coefficient, and IoU/Jaccard index when available. For metrics that were not reported or were not directly comparable, the corresponding entries are marked with “—”. Therefore, Table 8 should be interpreted as a cross-study comparative summary rather than a strictly controlled head-to-head benchmark.

As shown in Table 8, the proposed ensemble framework achieves competitive performance compared with both conventional segmentation baselines and recent state-of-the-art architectures. Compared with earlier methods such as MultiresUnet, SLNet, and R2U-Net, the proposed model provides improved segmentation performance on the selected LiTS17 subset. Recent models such as SBM–Attention U-Net, Improved SwinUNet, DiNA-SwinUNet, and RMAU-Net demonstrate strong performance by incorporating attention mechanisms, transformer-based global context modeling, residual learning, and multi-scale feature representation. In comparison, the proposed framework adopts a complementary ensemble design that integrates an improved U-Net for local, boundary-sensitive, and pixel-level feature extraction with a Graph U-Net for modeling spatial relationships among SLIC-derived superpixel regions. The high accuracy and sensitivity obtained by the proposed ensemble model indicate that the integration of convolutional feature learning and graph-based spatial relationship modeling can improve liver and liver tumor segmentation performance. For liver segmentation, the proposed ensemble achieved 99.20% accuracy, 99.3% sensitivity, and 90.80% Dice coefficient. For liver tumor segmentation, it achieved 98.10% accuracy, 98.4% sensitivity, and 90.30% Dice coefficient. These results suggest that the proposed hybrid ensemble architecture can effectively segment both liver and tumor regions in the selected LiTS17 subset. Nevertheless, the comparison should be interpreted with caution. Several compared methods were evaluated on different datasets, including LiTS, 3D-IRCADb, CHAOS, SLIVER07, and other liver CT datasets, while the proposed method was evaluated on a selected subset of LiTS17. Moreover, some studies reported only a subset of evaluation metrics. Therefore, Table 8 is intended to position the proposed model with respect to representative and recent segmentation frameworks rather than to claim strictly controlled head-to-head superiority over all state-of-the-art methods.

Beyond the quantitative comparison, the clinical relevance of the reported accuracy values should also be considered. From a clinical perspective, achieving 99.20% accuracy for liver segmentation and 98.10% accuracy for liver tumor segmentation suggests that the proposed framework has potential to support computer-assisted liver image analysis. In current clinical practice, liver and tumor assessment on CT images is mainly performed by radiologists using visual interpretation and, when needed, manual or semi-automatic contouring tools for lesion measurement, treatment planning, and follow-up assessment. However, manual segmentation is time-consuming, operator-dependent, and may vary between observers, particularly for tumors with weak boundaries, heterogeneous enhancement, low contrast, or noisy imaging conditions. Therefore, accurate automated segmentation may provide practical benefits by reducing the time required for contouring, improving consistency in liver and tumor volume estimation, and supporting downstream clinical tasks such as surgical planning, radiotherapy planning, treatment response assessment, and longitudinal monitoring. Nevertheless, the reported accuracy values should not be interpreted as evidence that the proposed model can replace radiologists or current clinical standards of care. Rather, the model should be considered as an auxiliary decision-support tool that may assist clinicians by providing rapid and consistent preliminary segmentation results. Since the present study was conducted on a selected subset of LiTS17 and did not include prospective, multi-center, or cross-dataset clinical validation, further evaluation on larger external datasets and real clinical workflows is required before practical deployment.

The proposed hybrid neural network architecture for automated liver and tumor segmentation is aligned with the broader development of biomedical engineering, medical imaging, computational healthcare, and AI-assisted decision-support systems. Recent studies have shown the importance of computational and experimental methods in colorectal cancer modeling [42], misclassification-aware infectious disease estimation [43], disease comorbidity graph analysis [44], spatial transcriptomics clustering using multimodal biomedical data [45], drug–cancer interaction and clinical translation [46], micro/nano biomaterial surface analysis [47], implant biocompatibility and drug delivery enhancement [48], perioperative anesthetic management [49], dental CBCT assessment [50], graph summarization using information-theoretic measures [51], cancer treatment optimization [52], ultrasound-based cellular proliferation analysis [53], cardiac SPECT reconstruction [54], health-economic modeling [55], emergency MRI diagnosis [56], neurovascular risk assessment [57], neuropsychiatric drug development [58], diagnostic ultrasound meta-analysis [59], rehabilitation balance assessment [60], spinal posture analysis in rehabilitation medicine [61], patient-centered healthcare design [62], extended reality in healthcare environments [63], mammographic tumor detection using deep learning [64], MRI-based autism diagnosis using machine vision [65], pathfinding optimization [66], digital service quality modeling [67], fusion-based surface roughness prediction [68], resilient network reconfiguration under cyberattacks [69], high-speed bearing optimization [70], knowledge transmission in educational systems [71], environmental vegetation monitoring [72], ecological impact assessment [73], passive architectural comfort systems [74], photocatalytic air purification materials [75], and high-temperature thermal radiation engineering [76]. Together, these studies highlight the increasing importance of robust, interpretable, and efficient computational models across medical, engineering, environmental, and infrastructure-related domains, supporting the relevance of the proposed improved U-Net and Graph U-Net ensemble framework for reliable CT-based liver and tumor segmentation. Recent studies further support the relevance of the proposed hybrid CT segmentation framework by emphasizing graph-based spatial modeling [77], multi-scale spatial analysis [78], integrated computational planning [79], AI-assisted decision-making [80], intelligent inference systems [81], efficient real-time computation [82], structured computational problem solving [83], AI-based learning frameworks [84], interdisciplinary model design [85], engineering-system development [86], spatial reasoning [87], optimization modeling [88], quantitative feature characterization [89], multi-factor decision frameworks [90], multimodal input processing [91], reliability-focused decision analysis [92], hierarchical structural modeling [93], predictive outcome modeling [94], visual pattern recognition [95], comparative feature identification [96], strategic systems modeling [97], classification-oriented analysis [98], biomedical validation [99], computational interpretation of biological complexity [100], translational clinical assessment [101], structural materials characterization [102], data-driven experimental analysis [103], morphology-based functional analysis [104], spatial surface-property modeling [105], quantitative image/data analytics [106], structural optimization [107], volumetric medical imaging [108], biomedical state modeling [109], clinical-radiological prediction [110], clinical outcome evaluation [111], and data-driven predictive inference [112], all of which align with the need for robust, interpretable, and efficient improved U-Net and Graph U-Net ensemble learning for automated liver and tumor segmentation on CT images.

This study, like many others, presents both strengths and limitations. One of the notable strengths of this research is the achievement of high segmentation accuracy for liver and liver tumor lesions, demonstrating the potential effectiveness of the proposed hybrid neural network architecture. Furthermore, the application of graph convolutional networks for modeling spatial relationships within medical images provides a useful framework for processing complex anatomical structures and capturing dependencies between image regions. However, this study also has several important limitations. First, the experiments were conducted on a selected subset of the LiTS17 dataset, including 9 CT volumes and 987 training images, rather than the full LiTS17 cohort. Although this subset allowed an initial evaluation of the proposed hybrid ensemble framework, the relatively small number of patient cases may limit the statistical strength and generalizability of the reported results. In particular, this limited subset may not fully represent the variability observed in real-world clinical CT data, including differences in scanner type, acquisition protocol, contrast phase, image quality, tumor size, tumor morphology, lesion appearance, and patient population. Therefore, the current findings should be interpreted as preliminary evidence of the model’s effectiveness rather than definitive proof of clinical generalizability. Second, cross-dataset validation was not conducted on independent public liver segmentation datasets such as 3DIRCADB, IRCADB01, or HCC-TACE-Seg. As a result, the robustness and transferability of the proposed model to unseen clinical data remain to be further investigated. Future work should evaluate the proposed framework using the complete LiTS17 dataset, larger multi-center cohorts, and independent external datasets to more comprehensively assess its generalization ability, robustness, and clinical reliability. Third, the proposed framework may introduce additional computational and memory overhead compared with a single convolutional segmentation network. Since the model integrates both an improved U-Net and a Graph U-Net within an ensemble architecture, training and inference require dense pixel-level convolutional feature extraction, SLIC superpixel generation, graph construction, graph convolution operations, and ensemble fusion. Consequently, the proposed model may require higher GPU memory, longer training time, and greater implementation complexity, particularly when processing high-resolution CT images or larger volumetric datasets. Although this additional computational cost is associated with improved segmentation accuracy and robustness under noisy imaging conditions, it may limit direct deployment in resource-constrained clinical environments. Future research should therefore investigate efficiency-oriented strategies, such as lightweight graph convolutional layers, model pruning, knowledge distillation, mixed-precision training, optimized SLIC implementation, and efficient inference pipelines. These improvements could help maintain the segmentation performance of the proposed framework while improving its practical efficiency for real-world computer-assisted clinical use.

In addition, future work should also consider the operational and infrastructure requirements of deploying computationally intensive AI models in healthcare facilities. Real-time CT segmentation may increase computational and energy demands, especially under uncertain imaging workloads. Therefore, data-driven uncertainty modeling, Monte Carlo simulation, and stochastic optimization can be explored to support cost-aware and energy-efficient deployment of AI-assisted clinical systems [113]. Moreover, reinforcement learning may be investigated for adaptive hyperparameter scheduling, learning-rate adjustment, inference scheduling, and resource-aware model execution, as tailored learning-rate strategies have shown potential for improving training stability and efficiency [114].

## 5. Conclusions

The proposed ensemble network, combining an improved U-Net and Graph U-Net architectures, demonstrates a highly effective approach for liver and liver tumor segmentation from CT images. Through careful pre-processing, including image augmentation and normalization, the system achieves remarkable performance in segmenting both liver and liver tumors with accuracy rates of 99.2% and 98.1%, respectively. The integration of advanced techniques such as SLIC for graph construction and the use of multi-layered convolutional strategies in U-Net ensures precision in detecting detailed anatomical features. Moreover, the network’s robustness is validated by its performance in noisy environments, where it outperforms other state-of-the-art methods, highlighting its practical applicability in clinical settings. Overall, the proposed method provides an efficient and reliable solution for medical image segmentation tasks, offering both high accuracy and resilience under varying conditions.

## Figures and Tables

**Table 1:** Details of the Graph-UShaped module of the Ensemble architecture.

Layer Number	Layer	Shape of Weight Vector	Layer Number	Layer	Shape of Weight Vector
1	First Graph Layer	[M1, Slices, Slices]	12	Graph Up-Pooling	[Slices/4]
2	Batch Normalization	[Slices]	13	First decoder part-Graph Layer	[M3, Slices/4, Slices/4]
3	Graph Pooling	[Slices/2]	14	Batch Normalization	[Slices/4]
4	Second Graph Layer	[M2, Slices/2, Slices/2]	15	Graph Up-Pooling	[Slices/2]
5	Batch Normalization	[Slices/2]	16	Second decoder part-Graph Layer	[M2, Slices/2, Slices/2]
6	Graph Pooling	[Slices/4]	17	Batch Normalization	[Slices/2]
7	Third Graph Layer	[M3, Slices/4, Slices/4]	18	Graph Up-Pooling	[Slices]
8	Batch Normalization	[Slices/4]	19	Third decoder part-Graph Layer	[M1, Slices, Slices]
9	Graph Pooling	[Slices/8]	20	Batch Normalization	[Slices]
10	Base Graph Layer	[M4, Slices/8, Slices/8]			
11	Batch Normalizayion	[Slices/8]			

**Table 2:** Details of the Improved UNet part of the Ensemble architecture.

Layer	Layer Name	Activation Function	Output Dimension	Size of Kernel	Stride Shape	Number of Kernels
1	Conv2-D	ReLU	(Batch, 256, 256, 64)	3 × 3	1 × 1	64
2	MaxPooling 2-D	-	(Batch, 128, 128, 64)			64
3	Conv2-D	ReLU	(Batch, 128, 128, 128)	3 × 3	1 × 1	128
4	Conv2-D	ReLU	(Batch, 128, 128, 128)	3 × 3	1 × 1	128
5	MaxPooling 2-D	-	(Batch, 64, 64, 128)			128
6	Conv2-D	ReLU	(Batch, 64, 64, 256)	3 × 3	1 × 1	256
7	Conv2-D	ReLU	(Batch, 64, 64, 256)	3 × 3	1 × 1	256
8	Conv2-D	ReLU	(Batch, 64, 64, 256)	3 × 3	1 × 1	256
9	MaxPooling 2-D	-	(Batch, 32, 32, 256)			256
10	Conv2-D	ReLU	(Batch, 32, 32, 512)	3 × 3	1 × 1	512
11	Conv2-D	ReLU	(Batch, 32, 32, 512)	3 × 3	1 × 1	512
12	Conv2-D	ReLU	(Batch, 32, 32, 512)	3 × 3	1 × 1	512
13	Conv2-D	ReLU	(Batch, 32, 32, 512)	3 × 3	1 × 1	512
14	MaxPooling 2-D	-	(Batch, 16, 16, 512)			512
15	Conv2-D	ReLU	(Batch, 16, 16, 1024)	3 × 3	1 × 1	1024
16	Conv2-D transpose	ReLU	(Batch, 32, 32, 512)	2 × 2	2 × 2	512
17	Concatenate		(Batch, 32, 32, 1024)			-
18	Conv2-D	ReLU	(Batch, 32, 32, 512)	2 × 2	2 × 2	512
19	Conv2-D	ReLU	(Batch, 32, 32, 512)	2 × 2	2 × 2	512
20	Conv2-D	ReLU	(Batch, 32, 32, 512)	2 × 2	2 × 2	512
21	Conv2-D	ReLU	(Batch, 32, 32, 512)	2 × 2	2 × 2	512
22	Conv2-D transpose	ReLU	(Batch, 64, 64, 256)	3 × 3	1 × 1	256
23	Concatenate		(Batch, 64, 64, 512)			-
24	Conv2-D	ReLU	(Batch, 64, 64, 256)	3 × 3	1 × 1	256
25	Conv2-D	ReLU	(Batch, 64, 64, 256)	2 × 2	2 × 2	256
26	Conv2-D	ReLU	(Batch, 64, 64, 256)	2 × 2	2 × 2	256
27	Conv 2-D transpose	ReLU	(Batch, 128, 128, 128)	3 × 3	1 × 1	128
28	Concatenate		(Batch, 128, 128, 256)			-
29	Conv2-D	ReLU	(Batch, 128, 128, 128)	3 × 3	1 × 1	128
30	Conv2-D	ReLU	(Batch, 128, 128, 128)	3 × 3	1 × 1	128
31	Conv2-D transpose	ReLU	(Batch, 256, 256, 64)	2 × 2	2 × 2	64
32	Concatenate		(Batch, 256, 256, 128)			-
33	Conv 2-D	ReLU	(Batch, 256, 256, 64)	3 × 3	1 × 1	64

**Table 3:** The details of optimal parameters.

Parameters	Search Scope	Optimal Value
**Optimizer of Improved U-net**	Adam	Adam
**Cost function of first part**	MAE, Dice Loss	Dice Loss
**SLIC**	10, 20, 40, 60	20
**Learning rate of first part of Ensemble Net**	0.01, 0.001, 0.0001	0.001
**M1, M2, M3, M4 of Graph Convolutional Network**	2, 3, 4	2
**Optimizer of Graph Unet**	Adam	Adam
**Learning rate of Graph Net**	0.0001, 0.00001	0.0001
**Number of Graph layers in decoder part**	2, 3, 4	3
**Number of steps in encoder part of the Improved u-net**	3, 4	4

**Table 4:** Segmentation results obtained based on the evaluation indicators used.

Lesion	Methods	Accuracy (%)	Sensitivity (%)	Dice-Coeff (%)	Mean-IoU (%)
**Liver**	Proposed Ensemble	99.2	99.3	90.8	89.9
**Liver Tumor**	Proposed Ensemble	98.1	98.4	90.3	89.4

**Table 5:** Segmentation results obtained based on different SLIC numbers.

Number of SLIC	Lesion	Accuracy (%)	Dice-Coeff (%)
10	Liver	90.8	83.4
20		99.2	90.8
30		99.2	90.8
10	Liver Tumor	86.6	81.5
20		98.1	90.3
30		98.1	90.3

**Table 6:** Hyperparameter sensitivity analysis of the proposed ensemble model.

Hyperparameter	Tested Values	Best Value	Observation
**Learning rate**	1 × 10^−5^, 5 × 10^−5^, 1 × 10^−4^, 5 × 10^−4^	1 × 10^−4^	Lower values slowed convergence; higher values reduced training stability
**Batch size**	4, 8, 16	8	Batch size 8 provided a balance between stable optimization and generalization
**Training epochs**	50, 100, 150	100	Performance improved up to 100 epochs and then showed limited additional gain
**Graph convolution layers**	1, 2, 3, 4	3	Three layers provided effective spatial modeling; deeper graph layers may cause over-smoothing

**Table 7:** Evaluation of the proposed model in the face of different noisy environments.

Methods	SNR: −4 dB	SNR: 0 dB	SNR: 10 dB	Noise Free
Proposed Improved Unet	81.5	82.3	86	95.6
Proposed Ensemble	85	86	89.2	99.2

**Table 8:** Evaluation of the proposed model with recent segmentation models.

Method	Dataset	Task	Accuracy (%)	Sensitivity (%)	Dice Coefficient (%)	IoU/Jaccard (%)	Ref.
**MultiresUnet**	Liver CT/LiTS-related	Liver/tumor segmentation	76.52	74.18	75.93	73.03	[33]
**SLNet**	Liver CT	Liver/tumor segmentation	—	64	54	—	[34]
**R2U-Net**	Medical image segmentation/liver CT	Liver/tumor segmentation	96.86	—	—	—	[35]
**MT-UNet++/UNet++-based model**	LITS2017	Liver segmentation	—	—	95.80	90.57	[36]
**SBM–Attention U-Net**	3Dircadb	Liver/tumor segmentation	—	—	93.77	88.89	[37]
**SBM–Attention U-Net**	LiTS	Liver/tumor segmentation	—	—	92.57	87.04	[37]
**SBM–Attention U-Net**	CHAOS	Liver segmentation	—	—	96.11	92.59	[37]
**Improved SwinUNet**	LiTS	Liver segmentation	—	—	95.59	91.55	[38]
**Improved SwinUNet**	LiTS	Liver tumor segmentation	—	—	76.14	61.47	[38]
**Improved SwinUNet**	3D-IRCADb	Liver segmentation	—	—	96.10	92.49	[38]
**Improved SwinUNet**	3D-IRCADb	Liver tumor segmentation	—	—	71.38	55.51	[38]
**DiNA-SwinUNet**	LiTS	Liver segmentation	—	—	97.50	95.12	[39]
**DiNA-SwinUNet**	SLIVER07	Liver segmentation	—	—	96.40	93.05	[39]
**RMAU-Net/ResUNet++-related model**	LiTS	Liver segmentation	—	—	95.52	91.42	[40]
**RMAU-Net/ResUNet++-related model**	LiTS	Liver tumor segmentation	—	—	76.16	61.50	[40]
**RMAU-Net/ResUNet++-related model**	3D-IRCADb	Liver segmentation	—	—	96.97	94.12	[40]
**RMAU-Net/ResUNet++-related model**	3D-IRCADb	Liver tumor segmentation	—	—	83.07	71.04	[40]
**LiTS benchmark top algorithms**	LiTS Challenge	Liver segmentation	—	—	96.30	92.86	[41]
**LiTS benchmark top algorithms**	LiTS Challenge	Liver tumor segmentation	—	—	67.40–73.90	50.83–58.60	[41]
**Proposed Improved U-Net**	Selected LiTS17 subset	Liver segmentation	95.60	—	—	—	This study
**Proposed Ensemble**	Selected LiTS17 subset	Liver segmentation	99.20	99.3	90.80	89.90	This study
**Proposed Ensemble**	Selected LiTS17 subset	Liver tumor segmentation	98.10	98.4	90.30	89.40	This study

## Data Availability

The dataset used in this study was derived from the publicly available LiTS17 dataset, which can be accessed through the official LiTS challenge repository in accordance with the dataset provider’s terms and conditions. In the present study, a selected subset of LiTS17 was used for model training and evaluation, as described in the Materials and Methods section. The source code and trained model weights are not publicly available at this stage due to author agreement, intellectual property considerations, and ongoing related research. However, detailed methodological descriptions, network configurations, preprocessing steps, training settings, and evaluation protocols have been provided in the manuscript to support reproducibility. Additional information may be made available from the corresponding author upon reasonable request, subject to approval by all authors.
